# Effects of plyometric training on softer vs. Harder surfaces on jump-related performance in rugby sevens players

**DOI:** 10.3389/fphys.2022.941675

**Published:** 2022-08-25

**Authors:** Alex Ojeda-Aravena, Jairo Azócar-Gallardo, Victor Campos-Uribe, Eduardo Báez-San Martín, Esteban Ariel Aedo-Muñoz, Tomás Herrera-Valenzuela

**Affiliations:** ^1^ IRyS Group, Physical Education School, Pontificia Universidad Católica de Valparaíso, Valparaíso, Chile; ^2^ Programa de Investigación en Deporte, Sociedad y Buen Vivir (DSBv), Universidad de Los Lagos, Osorno, Chile; ^3^ Departamento de Ciencias de la Actividad Física, Universidad de Los Lagos, Puerto Montt, Chile; ^4^ Facultad de Ciencias del Deporte, Toledo, Spain; ^5^ Programa de Vida Saludable, Actividad Física y Deporte, Undergraduate Studies, Universidad de Talca, Chile; ^6^ Carrera de Entrenador Deportivo, Escuela de Educación, Universidad Viña del Mar, Chile; ^7^ Departamento de Ciencias del Deporte, Facultad de Ciencias de la Actividad Física y del Deporte, Universidad de Playa Ancha, Valparaíso, Chile; ^8^ Departamento de Educación Física, Deportes y Recreación, Facultad de Artes y Educación Física, Santiago, Chile; ^9^ Universidad de Santiago de Chile (USACH), Facultad de Ciencias Médicas, Escuela de Ciencias de la Actividad Fisica, el Deporte y la Salud, Santiago, Chile

**Keywords:** plyometric exercise, strength training, team sports analysis, rugby 7s, training

## Abstract

This study aimed to compare jump-related performance after plyometric training on harder vs. softer surfaces in rugby sevens players. Fourteen players were randomly assigned to the harder surface group (H-G, *n* = 7) and softer surface group (S-G, *n* = 7). Three times per week, in the morning, the players performed plyometric training on different surfaces and strength training. Before and after the 4-week intervention period, squat jump (SJ), countermovement jump (CMJ), and CMJ with arms (CMJA) tests were performed to measure vertical jump displacement (d), rate of force development (r), and power (p). The main results indicated a significant improvement in S-G for CMJd (∆% = +8.2%; *p* = 0.029; ES = 0.59) and for CMJAp (∆% = +8.7%; *p* = 0.035; ES = 0.44). These improvements were significant compared to H-G for CMJAd (F_1,12_ = 8.50; *p* = 0.013; $\eta_{p}^{2}$ = 0.41; ES = 0.83) and CMJAp (F_1,12_ = 7.69; *p* = 0.017; $\eta_{p}^{2}$ = 0.39; ES = 0.79). This study reveals that performance related to the counter movement jump with arms on softer surfaces after 4-week plyometric training improved vertical jump displacement and lower body power in rugby sevens players.

## Introduction

Rugby sevens is an Olympic contact sport in which players (backs and forwards) need to express the ability to produce maximum muscle power, speed, and acceleration in the different motor actions during the game (e.g., jumping, running, tackling, changing direction) (Ross, Gill, & Cronin, 2015; Ross, Gill, Cronin, & Malcata, 2015; Loturco et al., 2017; Schuster et al., 2018; Watkins, Gill, et al., 2021). In this sense, they must develop specific physical abilities, including explosive strength and lower-body power. Such physical abilities are measured, among other tests, through squat jump (SJ), countermovement jump (CMJ), and CMJ with arms (CMJA) (Ross, Gill & Cronin, 2015; Schuster et al., 2018; Watkins, Gill, et al., 2021). For example, international-level rugby players display superior performance in absolute power by CMJ height (32%) than amateurs (Ross, Gill, & Cronin, 2015). Furthermore, in the CMJ, absolute power is reported between backs (*n* = 37) and forwards (*n* = 28) in absolute power (backs = 7,113 ± 1624 W vs. forwards = 7,966 ± 1841 W; −12%; effect size = 0.50) and relative power (back = 79.6 ± 13.8 W kg^−1^ vs. forwards = 81.8 ± 17.3 W kg^−1^; +1.9%; effect size = 0.10) (Ross, Gill, & Cronin, 2015). Thus, it is interesting that CMJ performance is positively associated with specific offensive and defensive actions, such as effective attack and defensive ruck during the game (Ross, Gill, Cronin, et al., 2015).

Based on the above, players are always looking for ways to bridge the gap between the strength gained in the gym and functional competition performance (Watkins, Storey, McGuigan, & Gill, 2021). In this sense, resistance training produces beneficial effects in rugby players in a one-repetition maximum (Crewther, Heke, & Keogh, 2013), in CMJ unloaded (Gathercole, Sporer, & Stellingwerff, 2015) and loaded (Weakley et al., 2021). Specifically, vertical plyometric training demonstrates positive effects on the rate of force development using isokinetic measures and SJ in young male elite rugby players (*n* = 11; age, 23.5 ± 0.9 years; height 173 ± 4.8 cm) (Cadore et al., 2013). Additionally, 3 weeks of reverse plyometric training focused on vertical and horizontal exercises with 12-day washout and low volume (40–60 ground contacts per session) in male rugby players (body mass = 102.6 ± 16.4 kg; height = 183.9 ± 6.9 cm; age = 19.8 ± 2.2 years) was effective in improving the linear sprint force-velocity profile. Particularly, the horizontal/vertical training group improved sprint performance, while the vertical/horizontal training group maintained sprint performance (Watkins, Gill, et al., 2021). Another study determined the magnitude of the effect of combined training (weightlifting-derived exercises, plyometric actions, and ballistic exercises) in rugby players distributed according to their different strength levels (stronger: one-repetition maximum squat = 2.01 ± 0.15 kg⋅BM^−1^; weaker: 1.20 ± 0.20 kg⋅BM^−1^). The authors reported that the preexisting strength level influenced these effects after 10 weeks. Specifically, the stronger group displayed a greater change in peak velocity at mid-test but not post-test when compared to the weaker participants, and only the stronger group displayed increases in muscle activation (James et al., 2018). Plyometric training effectively improves lower-body explosive power and dynamic athletic performance, particularly in movements involving the stretch-shortening cycle (Markovic, Jukic, Milanovic, & Metikos, 2007; Turner, 2009). Plyometric training emphasizes jumping exercises (vertical and horizontal) with bodyweight using the Stretch-shortening cycle as an effective neuromuscular stimulus that does not require a large physical place or expensive equipment (Ramírez-Campillo, Andrade, & Izquierdo, 2013). Moreover, this training method induces positive physiological adaptations related to the force–time profile and muscle pennation angle fibres (Palma-Muñoz et al., 2021).

These adaptations or improvements can be influenced by the hardness (i.e., the coefficient of restitution) of the type of surface on that player’s train (Ramírez-Campillo et al., 2013). In this regard, for example, Ramirez-Campillo et al. (2013) analysed the effects of different training volumes (moderate and high volume; 120 and 240 jumps, respectively) and surfaces (hard or gym floor vs. soft mat or track and field on a gym floor) of jump-based plyometric training (40-cm drop jump exercises) on physical performance in college students (*n* = 29; age = 16.89 ± 0.85 years) after 7 weeks. The main results displayed benefits in SJ height in the moderate-volume group, reductions in CMJ height in both groups, increases in the moderate-volume hard surface group, and a high-volume soft surface group in 20-cm drop jump performance. Similarly, Ramírez-Campillo et al. (2020) recently investigated the effects of plyometric jump training after 8 weeks on combined surfaces (grass, soil, sand, wood, gym carpet) vs. plyometric training on a single surface (grass) on physical performance (i.e., maximal kicking velocity, change of direction speed tasks, the 20-cm drop jump, muscle power, speed) in young soccer players (*n* = 23; age range = 11–14 years) during 8 weeks. The authors revealed that both groups improved CMJ height and long standing jumps. Although only the group that used different surfaces obtained positive changes in the components mentioned above (Ramirez-Campillo et al., 2020). On the other hand, Çimenli et al. (2016) applied 8 weeks of plyometric training on different surfaces (wooden vs. synthetic surface) in male volleyball players (*n* = 36; age range 18–24 years) on vertical, horizontal, unilateral, and bilateral jump performance. The authors reported positive changes in both surfaces in the majority of the measures analysed and concluded that the type of surface used did not influence plyometric training adaptations. Additionally, Ahmadi et al. (2021) evaluated the effect of 8 weeks of plyometric jump training on a soft surface (sand) vs. a hard surface (regular volleyball rigid surface) in female volleyball players (*n* = 22; age ∼22 years, height = ∼167 cm; body mass = ∼58 kg) on physical performance, including CMJ height and 20-cm drop jump. The results showed that both groups improved CMJ height, CMJ reactive strength index, and CMJ velocity-take off. However, the 20-cm drop jump height improved in the hard-surface group and the CMJ peak force in the soft-surface group (Ahmadi et al., 2021).

Therefore, it remains unclear whether chronic plyometric training on a particular surface confers more significant benefits to physical performance. It is one of the least studied aspects of plyometric training (Ramirez-Campill, et al., 2018a) and is limited primarily to understanding of the acute response (Pereira et al., 2021); consequently, it merits further study. In addition, physical adaptations to plyometric training would be influenced in a specific manner considering the exercise nature (i.e., drop jump vs. CMJ) depending on the surface used. Consequently, based on previous studies (Ramírez-Campillo et al., 2013; Çimenli et al., 2016; Ramirez-Campillo et al., 2020; Ahmadi et al., 2021), the hypothesis is that jump-related performance in rugby players will improve significantly after 4 weeks of plyometric training, although to a greater magnitude on softer vs. harder surfaces. Therefore, this study aimed to compare jump-related performance after plyometric training on harder vs. softer surfaces in rugby sevens players.

## Materials and methods

### Subjects

**FIGURE 1:**
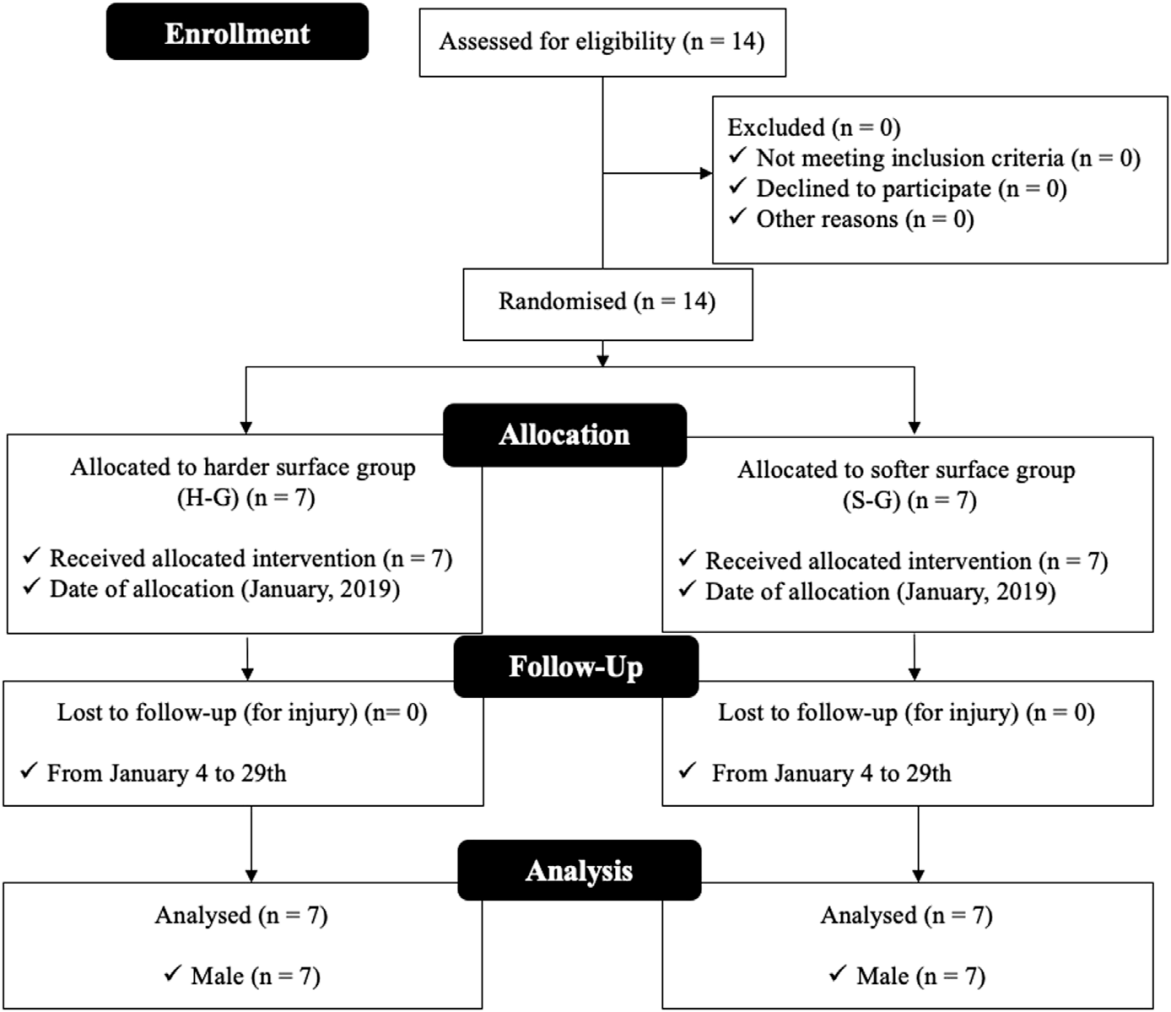
Flowchart of study.

In this quasi-experimental randomized parallel study design, male adult rugby sevens players (*n* = 14 males; age 23.6 ± 2.8 years; stature 171.5 ± 5.5 cm; body mass 73.3 ± 10 kg; fat mass percentage 14.4 ± 3.5%) who compete annually in national tournaments participated in this study. Players were randomly allocated into the harder surface group (H-G), *n* = 7 (23.2 ± 2.1 years; 171.1 ± 4.8 cm; 72.1 ± 10.2 kg; 13.3 ± 4.1%), and into the softer surface group (S-G), *n* = 7 (24.2 ± 3.2 years; 171.9 ± 5.2 cm; 74.5 ± 9.2 kg; 11.1 ± 7.1%) (Figure 1). The inclusion criteria for participation in this study were as follows: 1) More than 3 years of playing rugby sevens; 2) no history of illness or medication; 3) have not suffered injuries or fractures for at least the last 6 months; 4) train at least five times a week; and 5) prepare physically for tournaments organized by the National Rugby Sports Federation. All participants were informed in detail of the study’s procedures, benefits, and potential risks. The local ethics committee approved this study, and all participants provided written informed consent before the beginning of the study.

### Procedures

*A priori*, the sample size was determined using G*Power software (version 3.1.9.6; University of Düsseldorf, Düsseldorf, Germany) (Ahmadi et al., 2021). The *a priori* statistical power analysis performed included the following variables: effect size f: 5.8974 based on the effects of plyometric jump training and training surface on CMJp (Ahmadi et al., 2021): alpha error: 0.05; the correlation between groups: 0.5; desired power (1-ß error): 0.80. The *a priori* power analysis results indicated that a minimum of eight participants would be required to obtain the statistical significance of CMJp.

The assessments were performed on two nonconsecutive days. The first visit to the laboratory was made to familiarize the players with the jump tests and collect anthropometric data (age, stature, percentage of fat mass). After 48 h, the second visit was to assess jump tests (SJ, CMJ, and CMJA). The players were randomly assigned and distributed into H-G and S-G groups. Randomization (simple) was generated electronically (www.randomizer.org) and blinded before assigning study groups. The principal researcher coordinated the allocation sequence. Before and after the 4-week intervention period, the assessments were completed in the same order and at the same time of day (between 10:00 a.m. and 1:00 p.m.) with the same sports clothing and applied by the same professional. The professional responsible for the jump measurement tests were blinded to the assignment of the training groups. The players were previously instructed to 1) sleep well (≥ 8 hours) before each testing day, 2) maintain regular eating habits, 3) not consume energy drinks or any stimulant drugs before the assessments, and 4) use the same shoes in the assessments and during the intervention. The pre- and post-assessments were realized on the same surface (a synthetic gymnasium floor on that H-G trained). Before performing vertical jump tests, players performed a 10-min warm-up (submaximal sprints with a change of direction and 20 vertical and 10 horizontal jumps), joint mobility, dynamic flexibility, and three submaximal jump and sprint attempts.

### Procedures

#### Training program

The study was conducted within the precompetitive period during the 4 weeks of 2019. The mesocycle program consisted of strength, technical and tactical training sessions (Table 1 for details). Two training sessions were conducted thrice a week (Monday, Wednesday, and Friday). During the morning, from 10:00 to 11:00, a 1-h plyometric program was performed on softer vs. harder surfaces. The harder surface was a Pulastic synthetic gym floor, and the softer surface was three 12 cm Bronson gym carpets mounted on a Pulastic synthetic gym floor. During the afternoon, from 17:00 to 18:00, strength training was performed for 1 h. On Tuesday, Thursday, and Saturday, the players performed technical and tactical work for 1 h and a half. Exercise intensity was monitored using the Borg perceived exertion scale (RPE; 0-10).

**TABLE 1:** Description of the strength and plyometric volume distribution training program.

Day	Monday				Wednesday				Friday			
Week	1	2	3	4	1	2	3	4	1	2	3	4
Plyometric training volume												
SJ with arms	4/10	4/10	4/10	4/10					4/10	4/10	4/10	3/10
CMJA					5/10	6/10	4/10	1/10				
Slide skate					7/10	8/10	5/10	3/10				
Rebound jump	5/10	6/10	8/10	8/10					6/10	8/10	7/10	6/10
Hurdles rebound jump (40–60 cm × 1 m)					10/12	10/14	8/10	4/10				
Bulgarian squat jump	4/10	4/10	5/10	5/10					4/10	5/10	5/10	4/10
Strength, power and change of direction with ball	✓	✓	✓	✓	✓	✓	✓	✓	✓	✓	✓	✓
Strength training volume												
Hang clean	2/6/60	3/6/60	3/6/70	2/6/50	—	—	—	—	2/8/40	3/8/30	3/8/30	—
Bench Press	—	—	—	—	3/6/50	4/6/60	3/6/70	2/6/40	—	—	—	—
Barbell Row	3/6/50	4/6/60	3/8/60	2/6/40	—	—	—	—	3/6/50	4/6/50	3/6/40	—
Barbell Lunge	—	—	—	—	2/8/50	3/6/60	2/8/40	2/8/40	—	—	—	—

Values expressed as sets/repetitions ✓, exercises realised; —means, no exercise ;CMJA, Countermovement jump with arms; SJ, Squat Jump. The values are expressed as sets/repetitions/1-RM, percentage; -: no exercise.

### Plyometric training program

Plyometric training sessions included a warm-up (15 min) consisting of light jogging around a wood-surface gym (∼ 5 min), dynamic stretching (∼ 5 min), agility ladder exercises, and core exercises (planks: Three sets of 30 s with a 1-min pause between each set). Subsequently, players were immediately distributed into the H-G and S-G groups. Both groups performed plyometric training on their corresponding surfaces. Both groups performed four exercises per training session with 3 min and 5 min of recovery between each type of exercise during plyometric training. Experienced professionals verbally stimulated the players for all jumps and provided feedback on the movement pattern. Players were previously instructed to perform each exercise maximally (RPE-10). The total volume of weekly jumps was 510 jumps during the first week, 590 jumps during the second week, 500 jumps during the third week, and 380 jumps during the fourth week.

### Strength training program

Strength training sessions included a standardized warm-up (∼15 min) that included joint mobility and dynamic flexibility. After that, the players performed four exercises during the session (Table 1) with a 3-min recovery between each set and 5 min between each exercise. The 1-h session was then ended with continuous change of direction exercises (RPE 5-6). The players performed a total weekly volume of 104 repetitions at 40–60% of a one-repetition maximum (1-RM) during the first week, 132 repetitions at 30–60% of 1-RM during the second week, 118 repetitions at 30–70% of 1-RM in the third week and 52 at 40–50% of 1-RM in the fourth week.

### Anthropometric and body composition

Body mass (kg) and stature (cm) were determined using a mechanical column scale (Brand: SECA Model 700). Additionally, the fat mass percentage (%) was assessed using bioimpedance (OMRON-Model BF306) after 8 hours of fasting and without at least 48 h of exercise.

### Jump performance

The SJ, CMJ without arms, and CMJ with arms (CMJA) tests were assessed using a force platform (Bertec 4060-05 triaxial system, Bertec Corp., Columbus, Ohio, United States), reporting values in Newtons at 1,000 Hz. The displacement (cm; d), rate of force development (N.s^−1^; r), and relative power (W.kg^−1^; p) were recorded using standard protocols (Ramírez-Campillo et al., 2013). After recovery of two to 3 minutes, players repeated the same sequence. Each participant completed three trials with 2 min of recovery between attempts using the best attempts for subsequent statistical analysis. Between each assessment, passive recovery was ensured for 10 min.

### Statistical analysis

The data are presented as the mean ± standard deviation. A Shapiro–Wilk normality test was performed to confirm that all data were normally distributed, and the Levene’s test was used to confirm the data homoscedasticity. To determine possible differences in baseline between groups, an unpaired *t* test was executed. Subsequently, repeated-measures ANOVA variance analysis of the time factor (pre-post intervention) by the group factor (S-G and H-G) was performed. Where any significant differences were found, post hoc pairwise comparisons (Bonferroni adjusted) were used to examine where the differences lay. Cohen’s d was calculated as an effect size measure (ES) (Rhea, 2004). Threshold values for Cohen’s d ES statistics were <0.20 [trivial], 0.20 [small], 0.60, [moderate], 1.20 [large], 2.0 [very large], and 4.0 [extremely large] (Hopkins, Marshall, Batterham, & Hanin, 2009). In addition, the players’ interindividual responses were classified as responders (Rs) and nonresponders (NRs) and defined as individuals who could not demonstrate an increase or decrease (in terms of beneficial changes) in physical fitness that was greater than twice the technical error (TE) away from zero. The TE was calculated using a previously established equation (Bonafiglia et al., 2016). Each fitness assessment was performed before and after the intervention to calculate the TE for this study. A change in TE more than twice represented a high probability (i.e., 12:1 odds) that the observed response was a true physiological adaptation beyond what could have been expected due to technical and/or biological variability. Therefore, TE was the following [SJd, 2.05 (cm) × 2; SJr 1995 (N.s^−1^) × 2; SJp, 1.74 (W.kg^−1^) × 2; CMJd, 2.96 (cm) × 2; CMJr, 1,311.7 (N.s^−1^); CMJp, 2.00 (W.kg^−1^) × 2; CMJAd, 2.48 (cm) × 2; CMJAr, 1,656.6 (N.s^−1^) × 2 CMJAp 2.45 (W.kg^−1^) × 2] (Ramirez-Campillo, et al., 2018b). Furthermore, the Chi-square test (X^2^) was used to compare groups of subjects who were in the 2 × TE calculated in each outcome (NRs) or exceeded the TE or Rs two times. All assessments had an acceptable intra-class reliability (*r* > 0.90) ranging from 0.92 to 0.98. The statistical significance level used was set to *p* < 0.05. All statistical analyses were performed using GraphPad PRISM (version 6.0, San Diego, California).

## Results

According to the PEDro (Maher, Sherrington, Herbert, Moseley, & Elkins, 2003) and TESTEX (Smart et al., 2015) scales, we complied with the eligibility criteria (e.g., ≥ 3 years of experience in national rugby leagues), random allocation (e.g., randomly introduced to plyometric training with a harder surface or plyometric training with a harder surface), allocation concealment (e.g., players were unaware of which group they would be allocated to, at the time of their consent), intergroup homogeneity (e.g., no baseline differences between groups in measures), participation ≥85% (e.g., all players completed the study), intention to treat analysis (e.g., all players received training condition), between-group comparison (e.g., ANOVA was applied), measure of variability (e.g., standard deviation and interindividual analysis reported), attendance reported (e.g., the compliance with training was >90%), exercise intensity controlled (e.g., both groups instructed to perform the plyometric jumping exercises at maximum effort), and exercise volume/energy expended controlled (e.g., the plyometric training decreased volume per week and strength increase repetitions However, we were unable to comply with the blinding of players, blinding of professionals, blinding of assessors, activity monitoring of groups outside the intervention, and regular rugby training sessions (other than the total number of sessions and minutes of training).

### Results of the analysed factors

The data were normally distributed, and no significant differences were reported between the baseline analysed variables (*p* < 0.05). Table 2 describes in detail the measures analysed. We found a significant improvement in the S-G for CMJAd (∆% = +8.2%; *p* = 0.029; ES = 0.59) and CMJAp (∆% = +8.7%; *p* = 0.035; ES = 0.44) after plyometric training. These results were statistically significant in comparison with the H-G for CMJAd (F_1,12_ = 8.50; *p* = 0.013; $\eta_{p}^{2}$ = 0.41; ES = 0.83) and CMJAp (F_1,12_ = 7.69; *p* = 0.017; $\eta_{p}^{2}$ = 0.39; ES = 0.79). We did not find significant substantial increases in the other analysed measures.

**TABLE 2:** Measures of jump-related performance in both groups analysed (*n* = 14).

Measures	Group	Pre	Post	∆%	ES pre vs. Post	F1.12; P; η2p; ES group factor	F1.12; P; η2p; ES time factor	ES
SJd (cm)	H-G	34.6 ± 4.1	36.1 ± 5.03	+4.3	0.36	0.01; 0.910; 0.01; 0.10	3.61; 0.081; 0.23; 0.54	0.04
	S-G	34.3 ± 3.4	35.9 ± 4.7	+4.6	0.47			
SJr (N.s^−1^)	H-G	6.340 ± 1.644	6.068 ± 1.370	−4.2	0.16	0.12; 0.727; 0.11; 0.35	0.83; 0.379; 0.06; 0.25	0.24
	S-G	5.127 ± 1851	6.743 ± 2.736	+31.5	0.87			
SJp (W.kg^−1^)	H-G	31.2 ± 2.0	32.7 ± 4.2	+4.5	0.75	0.14; 0.708; 0.12; 0.36	5.03; 0.045: 0.29; 0.63	0.12
	S-G	30.2 ± 4.9	31.9 ± 6.2	+5.6	0.34			
CMJd (cm)	H-G	35.8 ± 3.2	37.8 ± 4.0	+5.2	0.62	0.07; 0.794; 0.00	2.20; 0.164; 0.15; 0.42	0.25
	S-G	36.0 ± 3.0	36.6 ± 4.8	+1.6	0.20			
CMJr (N.s^−1^)	H-G	6.787 ± 3.559	7.144 ± 2.928	+5.2	0.10	0.34; 0.571; 0.28; 0.62	2.94; 0.598; 0.24; 0.56	0.31
	S-G	7.871 ± 3.557	8.073 ± 3.405	+2.5	0.05			
CMJp (W.kg^−1^)	H-G	32.6 ± 3.8	34.3 ± 3.99	+5.2	0.65	0.24; 0.630; 0.02; 0.14	2.30; 0.155; 0.16; 0.43	0.29
	S-G	31.8 ± 5.5	32.4 ± 6.4	+1.8	0.10			
CMJAd (cm)	H-G	41.3 ± 5.4	43.5 ± 4.0	+5.3	0.40	0.06; 0.803; 0.05; 0.22	8.50; 0.013; 0.41; 0.83 *	0.24
	S-G	41.4 ± 5.9	44.8 ± 5.4 *	+8.2	0.59			
CMJAr (N.s^−1^)	H-G	6.289 ± 2.145	6.590 ± 2.357	+4.7	0.14	0.15; 0.704; 0.01; 0.10	2.11; 0.172; 0.15; 0.42	0.09
	S-G	5.272 ± 2.501	6.795 ± 2085	+22	0.60			
CMJAp (W.kg^−1^)	H-G	37.5 ± 4.9	39.6 ± 4.4	+5.6	0.42	0.68; 0,894; 0.02; 0.14	7.69; 0.017; 0.39; 0.79 *	0.01
	S-G	36.5 ± 7.2	39.7 ± 7.9 *	+8.7	0.44			

Data expressed as mean ± standard deviation. *, mean significant differences (*p* < 0.05). H-G, harder surface group; S-G, softer surface group; ∆%, percent delta differences; ES, d cohen effect size. F, F value; P, *p* value; $\eta_{p}^{2}$ , Eta squared partial. SJd, Squat jump displacement; SJr, Squat jump rate force of development; SJp, Squat jump power; CMJd, Countermovement jump displacement; CMJr, Countermovement jump rate force of development; CMJp, Countermovement jump power; CMJAd, Countermovement jump with arms displacement; CMJr, Countermovement jump with arms rate force of development; CMJp, Countermovement jump with arms power. Cm, centimeters; N/s^−1^, newton per second; W/kg^−1^, watts per kilogram.

### Interindividual responses

When analysing the interindividual responses of rugby players after plyometric training, 28.5% (*n* = 2) Rs was reported for SJd and SJp in the H-G, while 14.2% (*n* = 1) in the S-G for SJr and 28.5% (*n* = 2) Rs were reported, while no Rs was reported in the H-G. When analysing CMJd performance, 28.5% (*n* = 2) Rs and 14.2% (*n* = 1) Rs were reported. For CMJr and CMJp, 14.2% (*n* = 1) Rs was reported in both groups. For CMJAd and CMJAp, 28.5% (*n* = 2) and 42.8% (*n* = 3) of Rs were reported, respectively. For CMJAr in both groups, 14.2% (*n* = 1) of Rs were reported.

We found no statistical proportions between the Rs of both groups for SJd (X^2^ = 0.42; *p* = 0.514), SJr (X^2^ = 3.81; *p* = 0.050), CMJd (X^2^ = 0.00; *p* = 0.999), CMJr (X^2^ = 0.00; *p* = 0.999), CMJp (X^2^ = 0.42; *p* = 0.512), CMJAd (X^2^ = 1.07; *p* = 0.299), CMJAr (X^2^ = 0.00; *p* = 0.999) and CMJAp (X^2^ = 0.31; *p* = 0.557).

## Discussion

This study aimed to compare jump-related performance after plyometric training on harder vs. softer surfaces in rugby sevens players. The hypothesis was that jump-related performance in rugby sevens players would improve significantly after 4 weeks of plyometric training in both groups, although to a greater magnitude on the softer surface vs. the harder surface. The results showed that only the CMJA performance on the softer surface significantly improved displacement and relative power after training in S-G. In addition, there was a trend and effect size towards improved performance as a function of the measures analysed in both groups. The interindividual responses of the players after the plyometric training intervention revealed a range of 28.5–42% of responders in the group that used a softer surface. In contrast, only a range of 0–28% of responders in the group trained on the harder surface. Therefore, 4 weeks of plyometric training on a softer surface improved jump and power more than training on a harder surface in sevens rugby players.

Analysing our main results, the above evidence displays a positive effect following plyometric training on a softer surface. However, adaptations would be specific depending on the desired muscle strength ability, volume, and training specificity. Our results displayed a significant increase for CMJAd in S-G (+8.2%; ES small = 0.59) vs. H-G (+5.6%; ES small = 0.40) and for CMJp (+8.7%; ES small = 0.44 vs. +5.6%; ES small = 0.42, respectively), although with trivial to small differences between both groups for CMJAd and CMJAp (ES = 0.01; 0.24, respectively). In this regard, for example, Ahmadi et al. (2021) reported that after 8 weeks of plyometric training, volleyball players trained on a rigid surface (wood) (*n* = 9) and soft surface (sand) (*n* = 8) displayed that CMJ peak force performance was superior (*p* = 0.032) in the softer group (prevalue = 1,179 ± 230 N; postvalue = 1,457 ± 346 N; *p* < 0.001) than in the harder group (prevalue = 1,192 ± 222 N; postvalue = 1,236 ± 261 N; *p* < 0.53) (Ahmadi et al., 2021). In this regard, we report significant increases only for CMJAd and CMJp and not for CMJAr, where it is possible that the energy ground absorption (braking phase) (Chavda et al., 2018) from the softer surface during CMJA influenced improvements in the rate of force development. In addition, since this movement involves a slow stretching-shortening cycle, it is expected that emphasis on this specific measure will not be developed during exercise-based training, such as SJ with arms and CMJA. This aspect was noted by Ramirez-campillo et al. (2013), who reported significant decreases (*p* < 0.05) in CMJd on the hard surface with moderate volume (780 jumps) (prevalue = 31.9 ± 3.8 cm; postvalue = 30.1 ± 3.3 cm) and high volume (1,560 jumps) on the soft surface (prevalue = 35.1 ± 2.4 cm; postvalue = 33.6 ± 2.6 cm). In contrast, they documented increases in 20-cm drop jump performance in the high volume group on the soft surface (prevalue = 0.124 ± 0.03 cm ms^−1^; postvalue = 0.161 ± 0.02 cm ms^−1^) and in the group trained on a hard surface with moderate volume (prevalue = 0.124 ± 0.02 cm ms^−1^; postvalue = 0.161 ± 0.02 cm ms^−1^). Similarly, only in the moderate volume group on hard surfaces in drop jump 40 cm performance (prevalue = 0.112 ± 0.04 cm ms^−1^; postvalue = 0.142 ± 0.03 cm ms^−1^). The authors attributed the adaptations of these muscular characteristics to the specificity of the jumps applied (bounce drop jump) during training. Following this approach, Ramírez-Campillo et al. (2019) reported adaptations in CMJ height in both soccer players who performed plyometric training on hard surfaces (*n* = 8; prevalue = 34.7 ± 6.5 cm, postvalue 36.7 ± 6.7 cm; *p* < 0.001) and on combined surfaces (*n* = 8; prevalue = 31.7 ± 8.1 cm, postvalue 35.1 ± 9.2 cm; *p* < 0.001) (wood, grass, cement, mats and earth), although to a greater magnitude on combined surfaces (*p* < 0.001; *d* = 0.65) (Ramírez-Campillo et al., 2020).

Therefore, according to Ramirez-Campillo et al. (2013, 2020), a higher training volume and surface combinations could moderate the effect of the type of surface used during plyometric training. Specifically, this study revealed that only CMJA performance produced positive adaptations in displacement and jumped power, probably due to the specificity of the type of jump with the motor actions performed during the game. In this sense, this is in the same direction as indicated by Ramírez-Campillo et al. (2018a) because the type of surface can affect the speed of the stretch-shortening cycle (e.g., fast vs. slow), implying different biomechanical and physiological effects and different adaptations. Proposed mechanisms that could improve jumping performance on softer surfaces such as sand would include increased muscle activity of the muscle groups, increased motor unit recruitment, and neurological drive, reduced joint impact on tendons, and muscle-tendon unit stiffness (Pereira et al., 2021).

However, it is important to note that this study is not without limitations. Although the responses from two different surfaces were compared, they are not the surfaces typically trained by rugby sevens players. Therefore, future studies could include training on grass surfaces. They could also analyse the physiological adaptations of plyometric training on different surfaces, including hamstring stiffness applying movement involving a rapid stretch-shortening cycle (i.e., drop jump). Furthermore, it is necessary to mention that this is a quasi-experimental study, so the observed effects were not compared with a control group and should be corroborated with experimental studies.

Considering the above, this is the first study that analysed the responses to jump-related performance after 4 weeks of plyometric training using different surfaces in rugby sevens players. Particularly, it focused on exploring the effect of the surface at the chronic level on jump-related performance, one of the least studied aspects of plyometric training, according to Ramírez-Campillo et al. (2018b) in their recent scoping review. This was also noted by Pereira et al. (2021). In practical terms, coaches could use softer surfaces in sports with similar characteristics when weather conditions are adverse to reduce the probability of injury and optimize physical adaptations related to jumping in plyometric training. In addition, they could use a softer surface when they need to develop muscle power in the lower extremities. On the other hand, harder surfaces seem to effectively develop a fast stretch-shortening cycle, even though this must be corroborated.

## Conclusion

In conclusion, this study reveals that performance related to the countermovement jump with arms on softer surfaces after 4 weeks of plyometric training improved vertical jump displacement and lower body power in rugby sevens players.

## Data Availability

The raw data supporting the conclusion of this article will be made available by the authors, without undue reservation.

## References

[B1] Ahmadi M. Nobari H. Ramirez-Campillo R. Pérez-Gómez J. Ribeiro A. L. de A. Martínez-Rodríguez A. (2021). Effects of plyometric jump training in sand or rigid surface on jump-related biomechanical variables and physical fitness in female volleyball players. Int. J. Environ. Res. Public Health 18 (24), 13093. 10.3390/ijerph182413093 PubMed Abstract | 10.3390/ijerph182413093 | Google Scholar 34948702 PMC8701300

[B2] Bonafiglia J. T. Rotundo M. P. Whittall J. P. Scribbans T. D. Graham R. B. Gurd B. J. (2016). Inter-individual variability in the adaptive responses to endurance and sprint interval training: A randomized crossover study. PloS One 11 (12), e0167790. 10.1371/journal.pone.0167790 PubMed Abstract | 10.1371/journal.pone.0167790 | Google Scholar 27936084 PMC5147982

[B25] Cadore E. L. Pinheiro E. Izquierdo M. Correa C. S. Radaelli R. Martins J. B. (2013). Neuromuscular, hormonal, and metabolic responses to different plyometric training volumes in rugby players J. Strength Cond. Res. 27 (11), 3001–3010. 10.1519/JSC.0b013e31828c32de PubMed Abstract | 10.1519/JSC.0b013e31828c32de | Google Scholar 23442289

[B3] Chavda S. Bromley T. Jarvis P. Williams S. Bishop C. Turner A. N. (2018). Force-time characteristics of the countermovement jump: Analyzing the curve in excel. Strength Cond. J. 40 (2), 67–77. 10.1519/SSC.0000000000000353 10.1519/SSC.0000000000000353 | Google Scholar

[B4] Çimenli Ö. Koç H. Çimenli F. Kaçoglu C. (2016). Effect of an eight-week plyometric training on different surfaces on the jumping performance of male volleyball players. J. Phys. Educ. Sport 16 (1), 162. 10.7752/jpes.2016.01026 10.7752/jpes.2016.01026 | Google Scholar

[B5] Crewther B. T. Heke T. Keogh J. W. (2013). The effects of a resistance-training program on strength, body composition and baseline hormones in male athletes training concurrently for rugby union 7's. J. Sports Med. Phys. Fit. 53 (1), 34–41. PubMed Abstract | Google Scholar 23470909

[B6] Gathercole R. Sporer B. Stellingwerff T. (2015). Countermovement jump performance with increased training loads in elite female rugby athletes. Int. J. Sports Med. 36 (9), 722–728. 10.1055/s-0035-1547262 PubMed Abstract | 10.1055/s-0035-1547262 | Google Scholar 25831403

[B7] Hopkins W. Marshall S. Batterham A. Hanin J. (2009). Progressive statistics for studies in sports medicine and exercise science. Med. Sci. Sports Exerc. 41 (1), 3–13. 10.1249/MSS.0b013e31818cb278 PubMed Abstract | 10.1249/MSS.0b013e31818cb278 | Google Scholar 19092709

[B8] James L. P. Gregory Haff G. Kelly V. G. Connick M. J. Hoffman B. W. Beckman E. M. (2018). The impact of strength level on adaptations to combined weightlifting, plyometric, and ballistic training. Scand. J. Med. Sci. Sports 28 (5), 1494–1505. 10.1111/sms.13045 PubMed Abstract | 10.1111/sms.13045 | Google Scholar 29281133

[B9] Loturco I. Nakamura F. Y. Winckler C. Bragança J. R. da Fonseca R. A. Moraes-Filho J. (2017). Strength-power performance of visually impaired paralympic and olympic judo athletes from the Brazilian national team: A comparative study. J. Strength Cond. Res. 31 (3), 743–749. 10.1519/JSC.0000000000001525 PubMed Abstract | 10.1519/JSC.0000000000001525 | Google Scholar 27379958

[B10] Maher C. G. Sherrington C. Herbert R. D. Moseley A. M. Elkins M. (2003). Reliability of the PEDro scale for rating quality of randomized controlled trials. Phys. Ther. 83 (8), 713–721. 10.1093/ptj/83.8.713 PubMed Abstract | 10.1093/ptj/83.8.713 | Google Scholar 12882612

[B11] Markovic G. Jukic I. Milanovic D. Metikos D. (2007). Effects of sprint and plyometric training on muscle function and athletic performance. J. Strength Cond. Res. 21 (2), 543–549. 10.1519/R-19535.1 PubMed Abstract | 10.1519/R-19535.1 | Google Scholar 17530960

[B12] Palma-Muñoz I. Ramírez-Campillo R. Azocar-Gallardo J. Álvarez C. Asadi A. Moran J. (2021). Effects of progressed and nonprogressed volume-based overload plyometric training on components of physical fitness and body composition variables in youth male basketball players. J. Strength Cond. Res. 35 (6), 1642–1649. 10.1519/JSC.0000000000002950 PubMed Abstract | 10.1519/JSC.0000000000002950 | Google Scholar 34027922

[B13] Pereira L. A. Freitas T. T. Marín-Cascales E. Bishop C. McGuigan M. R. Loturco I. (2021). Effects of training on sand or hard surfaces on sprint and jump performance of team-sport players: A systematic review with meta-analysis. Strength Cond. J. 43 (3), 56–66. 10.1519/SSC.0000000000000634 10.1519/SSC.0000000000000634 | Google Scholar

[B14] Ramirez-Campillo R. Álvarez C. García-Hermoso A. Ramírez-Vélez R. Gentil P. Asadi A. (2018a). Methodological characteristics and future directions for plyometric jump training research: A scoping review. Sports Med. 48 (5), 1059–1081. 10.1007/s40279-018-0870-z PubMed Abstract | 10.1007/s40279-018-0870-z | Google Scholar 29470823

[B15] Ramirez-Campillo R. Álvarez C. García-Pinillos F. García-Ramos A. Loturco I. Chaabene H. (2020). Effects of combined surfaces vs. Single-surface plyometric training on soccer players’ physical fitness. J. Strength Cond. Res. 34 (9), 2644–2653. 10.1519/JSC.0000000000002929 PubMed Abstract | 10.1519/JSC.0000000000002929 | Google Scholar 30664111

[B16] Ramirez-Campillo R. Alvarez C. Gentil P. Moran J. García-Pinillos F. Alonso-Martínez A. M. (2018b). Inter-individual variability in responses to 7 Weeks of plyometric jump training in male youth soccer players. Front. Physiol. 9, 1156. 10.3389/fphys.2018.01156 PubMed Abstract | 10.3389/fphys.2018.01156 | Google Scholar 30177889 PMC6109752

[B17] Ramírez-Campillo R. Andrade D. C. Izquierdo M. (2013). Effects of plyometric training volume and training surface on explosive strength. J. Strength Cond. Res. 27 (10), 2714–2722. 10.1519/JSC.0b013e318280c9e9 PubMed Abstract | 10.1519/JSC.0b013e318280c9e9 | Google Scholar 23254550

[B18] Rhea M. R. (2004). Determining the magnitude of treatment effects in strength training research through the use of the effect size. J. Strength Cond. Res. 18 (4), 918–920. 10.1519/14403.1 PubMed Abstract | 10.1519/14403.1 | Google Scholar 15574101

[B19] Ross A. Gill N. Cronin J. Malcata R. (2015). The relationship between physical characteristics and match performance in rugby sevens. Eur. J. Sport Sci. 15 (6), 565–571. 10.1080/17461391.2015.1029983 PubMed Abstract | 10.1080/17461391.2015.1029983 | Google Scholar 25868066

[B20] Schuster J. Howells D. Robineau J. Couderc A. Natera A. Lumley N. (2018). Physical-preparation recommendations for elite rugby sevens performance. Int. J. Sports Physiol. Perform. 13 (3), 255–267. 10.1123/ijspp.2016-0728 PubMed Abstract | 10.1123/ijspp.2016-0728 | Google Scholar 28771098

[B21] Smart N. A. Waldron M. Ismail H. Giallauria F. Vigorito C. Cornelissen V. (2015). Validation of a new tool for the assessment of study quality and reporting in exercise training studies: Testex. Int. J. Evid. Based. Healthc. 13 (1), 9–18. 10.1097/XEB.0000000000000020 PubMed Abstract | 10.1097/XEB.0000000000000020 | Google Scholar 25734864

[B22] Turner A. N. (2009). Strength and conditioning for muay Thai athletes. Strength Cond. J. 31 (6), 78–92. 10.1519/SSC.0b013e3181b99603 10.1519/SSC.0b013e3181b99603 | Google Scholar

[B23] Watkins C. M. Gill N. D. Maunder E. Downes P. Young J. D. McGuigan M. R. (2021). The effect of low-volume preseason plyometric training on force-velocity profiles in semiprofessional rugby union players. J. Strength Cond. Res. 35 (3), 604–615. 10.1519/JSC.0000000000003917 PubMed Abstract | 10.1519/JSC.0000000000003917 | Google Scholar 33395182

[B24] Weakley J. J. S. Till K. Read D. B. Leduc C. Roe G. A. B. Phibbs P. J. (2021). Jump training in rugby union players: Barbell or hexagonal bar? J. Strength Cond. Res. 35 (3), 754–761. 10.1519/JSC.0000000000002742 PubMed Abstract | 10.1519/JSC.0000000000002742 | Google Scholar 29985223

